# Screening and Validation of LTBP1 as a Key Target of Oxymatrine in Inhibiting Cardiac Fibroblast Differentiation Under High Glucose Conditions: In Vitro and Bioinformatic Studies

**DOI:** 10.3390/ijms27083481

**Published:** 2026-04-13

**Authors:** Lianqing Tian, Shiquan Gan, Youqi Du, Chaowen Long, Churui Chang, Xiangchun Shen

**Affiliations:** 1 The State Key Laboratory of Functions and Applications of Medicinal Plants, Guizhou Medical University, No. 6 Ankang Avenue, Guian New District, Guiyang 561113, China; 2023110030636@stu.gmc.edu.cn (L.T.); ganshiquan@gmc.edu.cn (S.G.); 2023110030664@stu.gmc.edu.cn (Y.D.); 2023120030789@stu.gmc.edu.cn (C.L.); 2 The Department of Pharmacology of Materia Medica (The High Efficacy Application of Natural Medicinal Resources Engineering Center of Guizhou Province and The High Educational Key Laboratory of Guizhou Province for Natural Medicinal Pharmacology and Drug Ability), School of Pharmaceutical Sciences, Guizhou Medical University, No. 6 Ankang Avenue, Guian New District, Guiyang 561113, China; 3 The Key Laboratory of Optimal Utilization of Natural Medicine Resources (The Union Key Laboratory of Guiyang City, Guizhou Medical University), School of Pharmaceutical Sciences, Guizhou Medical University, No. 6 Ankang Avenue, Guian New District, Guiyang 561113, China

**Keywords:** diabetic cardiomyopathy, oxymatrine, LTBP1, molecular docking, MD simulations, bioinformatic study

## Abstract

Diabetic cardiomyopathy (DCM) features progressive fibrotic remodeling, but the shared molecular circuitry connecting diabetes mellitus (DM) to cardiomyopathy (CM) remains unclear. We integrated three DM- and three CM-related Gene Expression Omnibus (GEO) datasets and corrected batch effects with sva, verified by violin plots, principal component analysis (PCA), and silhouette coefficients computed on all common genes (DM: 0.9489 to −0.1016; CM: 0.9693 to −0.045; PC1/PC2 inter-batch differences abolished after normalization). Differential expression analysis identified 2562 DM Differentially expressed genes (DEGs) and 1414 CM DEGs, and their intersection yielded 91 common DEGs (51 upregulated, 40 downregulated). Protein–protein interaction (PPI) analysis prioritized 25 hub genes, whose enrichment profiles implicated insulin resistance/insulin signaling and adrenergic signaling in cardiomyocytes. TRRUST-based inference further defined a regulatory network centered on seven key genes (*HIF-1α*, *ACTN4*, *ABCB1*, *LTBP1*, *CLU*, *TIMP2*, and *MYH11*). To nominate a candidate target of oxymatrine (OMT), we performed docking and molecular dynamics (MD) simulations for representative complexes; OMT showed the most stable interaction with LTBP1, maintaining a consistently short pocket distance (~0.2 nm), the highest contact frequency, and the lowest MM/PBSA binding free energy (−15.32 kcal/mol), with favorable contributions dominated by van der Waals and nonpolar solvation terms. In primary cardiac fibroblasts (CFs), high glucose (HG, 30 mM glucose) induced proliferative and profibrotic activation, whereas OMT (0.4–0.8 mM) reduced HG-driven proliferation without detectable toxicity below 1.2 mM, suppressed FN, collagen I/III, and α-SMA expression, and inhibited migration. OMT also normalized HG-induced cell-cycle skewing by restoring G0/G1-phase occupancy and reducing S-phase entry, with effects comparable to metformin. Finally, HG increased LTBP1 expression and upregulated SMAD3/SMAD4, while OMT attenuated LTBP1 induction and suppressed downstream TGF-β/SMAD activation. Together, these data integrate cross-dataset transcriptomics with mechanistic validation to position LTBP1 as a putative antifibrotic node targeted by OMT, supporting inhibition of the LTBP1/TGF-β/SMAD axis as a candidate strategy to counter DCM-associated fibrosis.

## 1. Introduction

The global prevalence of diabetes mellitus (DM) has increased rapidly, resulting in progressive target-organ damage and substantially poorer clinical outcomes [1]. Among the cardiovascular complications of DM, diabetic cardiomyopathy (DCM) represents a major contributor to diabetes-related mortality, owing to its direct impairment of cardiac structure and function [2]. The European Society of Cardiology (ESC) defines DCM as diabetes-associated myocardial systolic and/or diastolic dysfunction occurring in the absence of overt coronary artery disease or hypertension [3]. DCM is characterized by extensive myocardial fibrosis, maladaptive cardiac remodeling and impaired diastolic relaxation, which subsequently progresses to systolic dysfunction and culminates in overt heart failure [4,5]. Myocardial fibrosis constitutes a central pathological hallmark of DCM, marked by profound disruption of extracellular matrix (ECM) homeostasis and serving as a key driver of adverse cardiac remodeling and heart failure progression [6]. Cardiac fibroblasts (CFs) play a pivotal role in maintaining ECM integrity under physiological conditions while also acting as principal effectors of pathological remodeling in disease states [7]. In response to pathological stimuli, CFs undergo activation, proliferation and phenotypic transition into myofibroblasts [8,9]. These activated cells secrete excessive amounts of collagen and other ECM components, thereby accelerating fibrotic deposition and contributing critically to myocardial stiffness and dysfunction [10]. The pathogenesis of DCM is multifactorial and complex. Chronic hyperglycemia induces oxidative stress, inflammation and endothelial dysfunction, which converge to promote CFs activation and fibrotic remodeling [11]. Although current therapeutic approaches, including renin–angiotensin–aldosterone system (RAAS) inhibitors and sodium–glucose cotransporter 2 (SGLT2) inhibitors, have shown efficacy in attenuating myocardial fibrosis and improving cardiac function in patients with diabetes [12,13], their overall impact remains limited. Given the intricate pathophysiology of DCM, there is a pressing need to develop novel therapeutic strategies that directly target fibroblast activation and ECM remodeling. Restoring the balance between ECM synthesis and degradation, and restraining aberrant CFs activation, is therefore essential for preventing and treating diabetes-associated cardiac dysfunction. Oxymatrine (OMT) is a quinolizidine alkaloid isolated from the dried roots of the traditional Chinese medicinal herb *Sophora flavescens* Aiton. It exhibits a broad spectrum of pharmacological activities, including anti-inflammatory, anti-oxidant, anti-tumor, and antiviral effects, as well as the regulation of metabolic processes [14,15,16,17,18]. Accumulating evidence indicates that OMT confers pronounced cardioprotective effects in experimental models, where it ameliorates heart failure, suppresses inflammatory responses and attenuates oxidative stress [19,20,21]. In addition, recent studies have reported hypoglycaemic and lipid-lowering effects of OMT in patients with DM [22]. However, the molecular mechanisms through which OMT regulates cardiac fibroblast activation during fibrogenesis remain poorly defined, and further investigation is required to elucidate its therapeutic potential in DCM.

Over the past several years, bioinformatics enrichment tools have become indispensable for the functional characterization of large gene sets, substantially advancing gene function analysis in high-throughput biological studies [23]. Molecular docking has likewise emerged as a powerful and widely used in silico approach in modern drug discovery, enabling the prediction of ligand–protein interactions through the estimation of binding affinities and the identification of potential binding sites and interaction modes, thereby providing mechanistic insights into ligand–target recognition [24]. However, docking results offer only a static representation of binding events. To overcome this limitation, molecular dynamics (MD) simulations are increasingly employed to investigate ligand-induced conformational changes and the dynamic behaviour of proteins during association and dissociation processes [25]. By capturing protein flexibility and dynamics, MD simulations provide a more comprehensive understanding of the relationship between protein structure, function and ligand binding. The integration of molecular docking with MD simulations enhances the reliability of in silico predictions by assessing the stability and dynamic properties of ligand–protein complexes over time.

In this study, we first identified potential therapeutic targets associated with DCM through integrative bioinformatics analyses. We then employed molecular docking and MD simulations to assess the binding modes and interaction stability between these targets and OMT. Furthermore, an in vitro high glucose (HG)-induced primary neonatal rat cardiac fibroblast model of DCM was established to validate target expression and to evaluate the effects of OMT intervention. Collectively, these findings provide novel mechanistic insights and suggest that LTBP1 may serve as a potential therapeutic target for the treatment of DCM.

## 2. Results

### 2.1. Batch Removal for Dataset Integration and Identification of Differentially Expressed Genes (DEGs)

**Figure 1:**
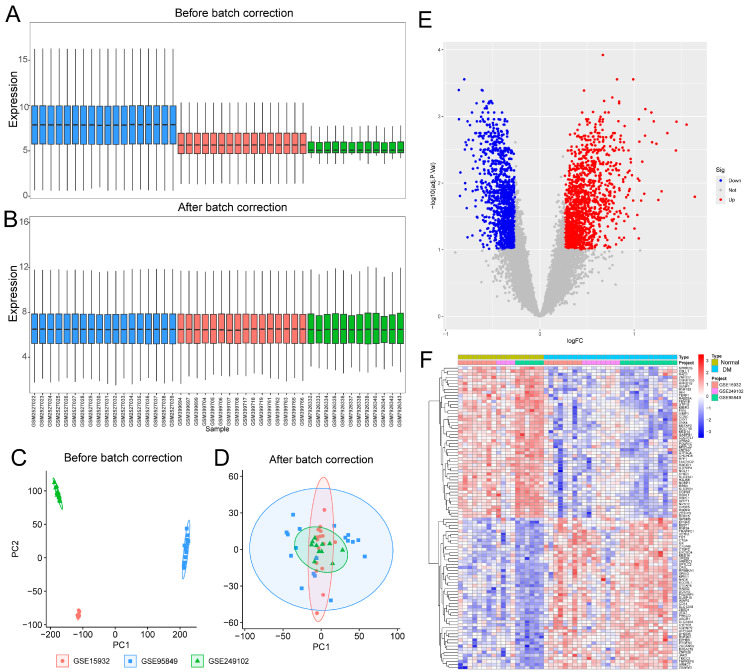
Batch effect removal and differential expression analysis in diabetes dataset integration. (**A**,**B**) Box plots before and after correction. (**C**,**D**) Principal component analysis (PCA) plots before and after correction. (**E**) Volcano plot of Differentially Expressed Genes (DEGs) in the diabetes mellitus (DM) and normal groups in the merged dataset. (**F**) Heatmap of DEGs in the DM and Normal groups in the merged dataset.

**Figure 2:**
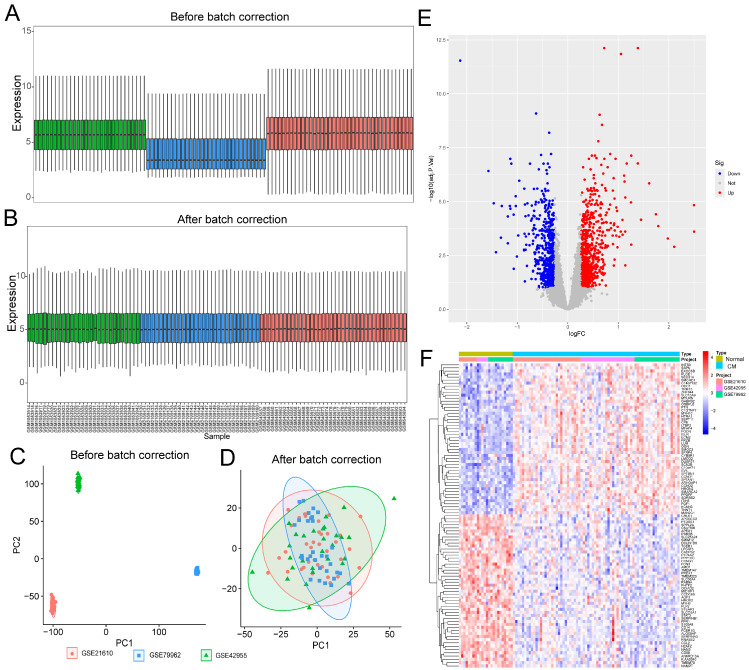
Batch effect removal and differential expression analysis in cardiomyopathy dataset integration. (**A**,**B**) Box plots before and after correction. (**C**,**D**) PCA plots before and after correction. (**E**) Volcano plot of DEGs in the cardiomyopathy (CM) and normal groups in the merged dataset. (**F**) Heatmap of DEGs in the CM and normal groups in the merged dataset.

Batch effects across DM-related datasets (GSE15932, GSE95849, and GSE249102) and CM-related datasets (GSE21610, GSE42955, and GSE79962) were corrected using the R package sva. The effectiveness of normalization was systematically evaluated by comparing sample distributions and clustering patterns before and after correction using violin plots and principal component analysis (PCA) (Figures S1 and S2; Figure 1A–D; Figure 2A–D). To ensure an unbiased and comprehensive assessment, PCA and silhouette coefficient analyses were performed on the complete set of all common genes across the integrated datasets rather than on pre-selected DEGs. Before correction, PC1 and PC2 scores showed significant inter-batch differences (DM: *p* < 0.001; CM: *p* < 0.001). These differences were effectively eliminated following normalization (DM: PC1, *p* = 0.998; PC2, *p* = 0.999; CM: PC1, *p* = 0.996; PC2, *p* = 0.993) (Figure S1A,B; Figure S2A,B). Consistently, PCA visualizations demonstrated pronounced batch-driven clustering prior to correction (DM silhouette coefficient = 0.9489; CM = 0.9693), which was substantially reduced after normalization (DM = −0.1016; CM = −0.045) (Figure S1C,D; Figure S2C,D), confirming effective removal of batch-associated variation. Subsequent differential expression analysis identified 2562 DEGs between DM patients and healthy controls (1331 upregulated and 1231 downregulated) and 1414 DEGs between CM patients and controls (780 upregulated and 634 downregulated) (Figure 1E,F; Figure 2E,F), thereby establishing a reliable foundation for downstream functional enrichment and pathway analyses.

### 2.2. Integrated Bioinformatics Analysis of Common DEGs (Co-DEGs) and Regulatory Networks in DCM

**Figure 3:**
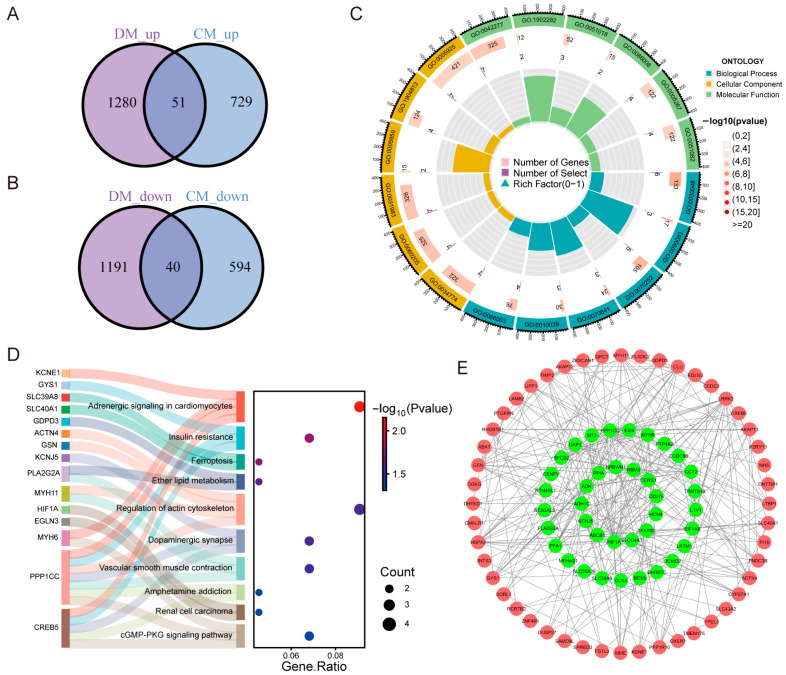
Integrated enrichment and network analysis of shared DEGs between DM and CM. (**A**,**B**) Venn diagrams of upregulated and downregulated genes in DM and CM. (**C**) Gene Ontology (GO) circle plot of DEGs. (**D**) Sankey diagram showing common DEGs (Co-DEGs) in diabetic cardiomyopathy (DCM) and their enriched Kyoto Encyclopedia of Genes and Genomes (KEGG) pathways, highlighting potential functional roles. (**E**) Protein–protein interaction (PPI) network of shared DEGs generated in Cytoscape, with core genes highlighted in green.

**Figure 4:**
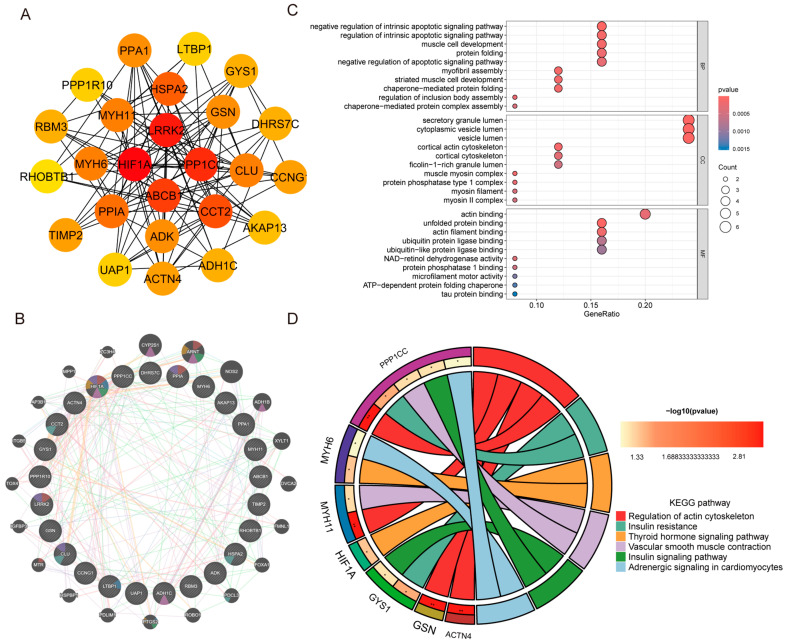
Functional association and enrichment analysis of the top 25 core genes. (**A**) Cytoscape visualization of the top 25 core genes. (**B**) GeneMANIA network of the core genes. Edge colors indicate different types of associations: predicted interactions (orange), physical interactions (pink), co-expression (light purple), pathway (light cyan), co-localization (light blue), genetic interactions (light green), and shared protein domains (light khaki). Colored sectors within the nodes represent major biological functions, including cellular response to oxidative stress (brick red), regulation of transforming growth factor beta production (blue), vascular endothelial growth factor production (green), hormone metabolic process (magenta), protein folding (teal), regulation of vascular endothelial growth factor production (golden yellow), and negative regulation of intrinsic apoptotic signaling pathway (purple). (**C**) GO enrichment analysis of the core genes. (**D**) The sectors on the left represent genes enriched in the pathways, whereas the sectors on the right represent significantly enriched KEGG pathways. Colored links indicate the associations between genes and their corresponding pathways. The color of the right-hand rectangles ranges from light yellow to red, representing increasing enrichment significance expressed as −log10(*p* value). Asterisks indicate the significance level, where ** denotes *p* < 0.01, and * denotes the remaining entries according to the current plotting script.

DEGs identified from the DM- and CM-related datasets were intersected, yielding 91 Co-DEGs, comprising 51 upregulated and 40 downregulated genes, which were visualized using Venn diagrams (Figure 3A,B). These shared transcriptional alterations suggest the existence of overlapping molecular mechanisms between DM and CM. To characterize the biological functions of these Co-DEGs, Gene Ontology (GO) and Kyoto Encyclopedia of Genes and Genomes (KEGG) pathway enrichment analyses were performed. GO enrichment analysis revealed the distribution of Co-DEGs across biological process (BP), cellular component (CC) and molecular function (MF) (Figure 3C). KEGG pathway analysis indicated that several genes, including HIF-1α and MYH11, were significantly enriched in multiple signaling pathways, such as renal cell carcinoma and regulation of the actin cytoskeleton (Figure 3D). To investigate the interactions among the 91 Co-DEGs, a Protein–Protein Interaction (PPI) network was constructed using the STRING database and visualized via Cytoscape (Figure 3E). Based on the topological node degree, the top 25 genes were identified as hub genes (Figure 4A). The functional relationships among these 25 hub genes were further mapped using the GeneMANIA platform, which predicted network associations including co-expression, physical interactions, and shared pathways (Figure 4B). Subsequent GO and KEGG enrichment analyses specific to these hub genes demonstrated significant enrichment in pathways directly relevant to metabolic and cardiac stress, specifically including insulin resistance, insulin signaling, and adrenergic signaling in cardiomyocytes (Figure 4C,D).

### 2.3. OMT Suppresses Cell Differentiation, Collagen Synthesis, and Migration in CFs Under HG Conditions

**Figure 5:**
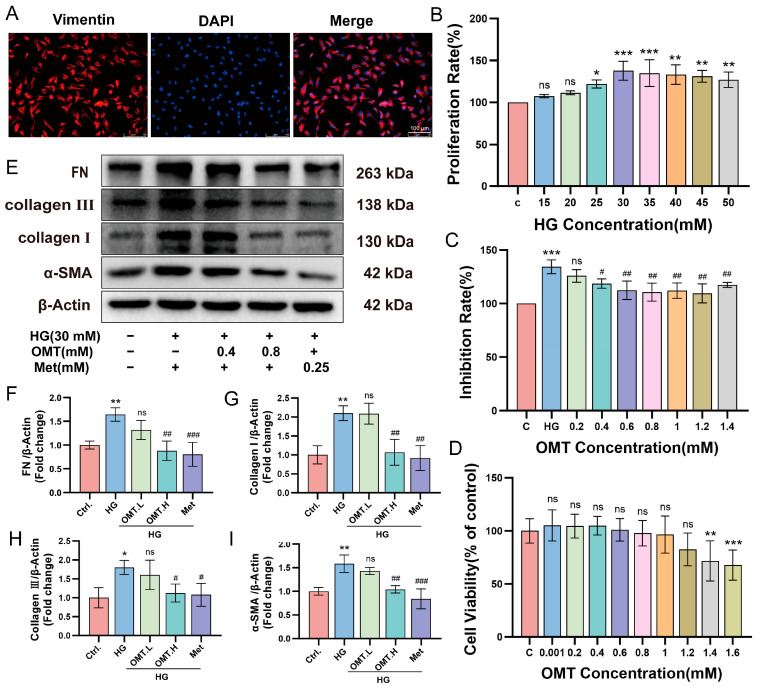
Oxymatrine (OMT) inhibits cell proliferation and regulates the expression levels of fibrosis-related proteins (*n* = 3). (**A**) Immunofluorescence Staining for Vimentin in Cardiac fibroblasts (CFs) Cells to Assess Purity. Vimentin is shown in red, and nuclei stained with DAPI are shown in blue. Scale bar: 100 µm. Images were captured using a 20× objective. (**B**) Optimization of high glucose (HG) Model Concentration. (**C**) Exploration of OMT Drug Concentration. (**D**) The evaluation of OMT cytotoxicity at different concentrations. (**E**–**I**) The expression level of fibrosis-related proteins after OMT pre-treatment. Data are presented as mean ± standard deviation (SD). * *p* < 0.05, ** *p* < 0.01, *** *p* < 0.001 vs. Ctrl, # *p* < 0.05, ## *p* < 0.01, ### *p* < 0.001 vs. HG; ns, not significant.

Sustained hyperglycemia disrupts cardiac metabolic homeostasis and, in severe cases, leads to myocardial injury. Cardiac damage activates CFs, promoting excessive proliferation and phenotypic trans-differentiation, thereby driving myocardial fibrosis [26]. To recapitulate key pathological features of DCM in vitro, we first verified the quality of the isolated cells. Immunofluorescence staining for the fibroblast-specific marker Vimentin confirmed that the purity of the isolated CFs was approximately 98% (Figure 5A). Subsequently, cells were exposed to a range of glucose concentrations (15–50 mM) for 24 h to determine the optimal stimulation condition. CF proliferative activity was significantly increased under high glucose conditions, with the most pronounced effect observed at 30 mM glucose (Figure 5B). Consequently, 30 mM glucose was selected as the HG condition for all subsequent experiments. Notably, treatment with OMT at concentrations of 0.4 and 0.8 mM markedly attenuated HG-induced CF proliferation (Figure 5C). Cytotoxicity assays further confirmed that OMT exerted no detectable toxic effects at concentrations below 1.2 mM (Figure 5D). We next examined the effects of OMT on HG-induced CFs activation and ECM production and migratory capacity. Exposure to HG markedly increased the expression of fibrotic markers, including FN, collagen I, collagen III and α-SMA, whereas OMT treatment significantly suppressed the upregulation of these proteins. Crucially, the positive control Met exhibited potent antifibrotic activity, as evidenced by the significant downregulation of these markers, thereby validating the reliability of our experimental model (Figure 5E–I). These findings indicate that OMT effectively inhibits HG-induced CFs activation and fibrogenic responses, with an efficacy comparable to that of Met.

**Figure 6:**
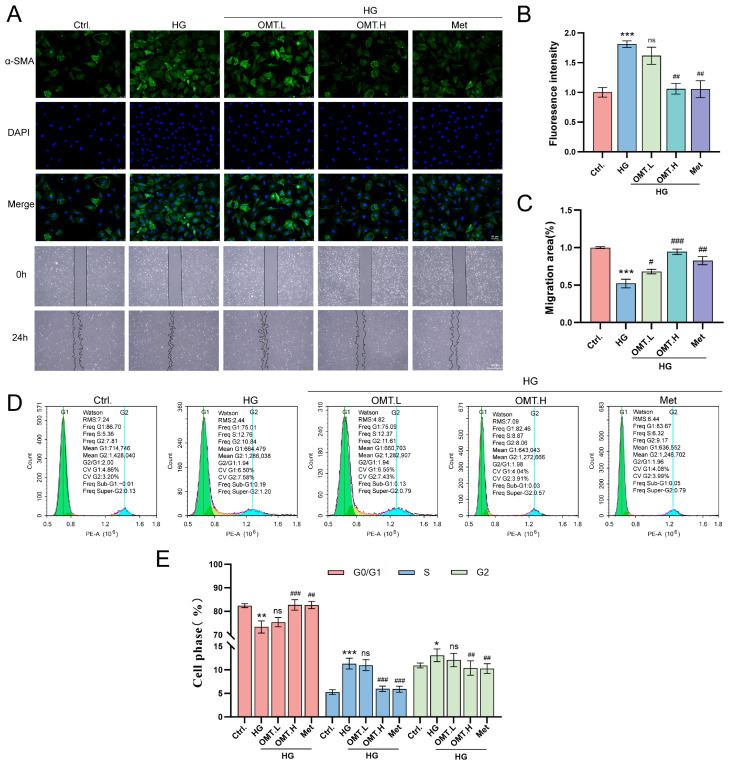
Effects of OMT on α-SMA expression, cell migration, and cell cycle progression in a HG-induced cardiac fibroblast model (*n* = 3). (**A**,**B**) Immunofluorescence staining demonstrates the expression levels of α-SMA under different experimental conditions, with α-SMA is shown in green, and nuclei stained with DAPI are shown in blue. Scale bar: 50 µm. Images were captured using a 40× objective. (**C**,**D**) Wound healing assay shows cell migration at 0 and 24 h after creating a scratch wound. Scale bar: 100 µm. Images were captured using a 20× objective. (**E**) The effect of oxidized picloram on cell cycle distribution was explored using flow cytometry analysis. Data are presented as mean ± SD. * *p* < 0.05, ** *p* < 0.01, *** *p* < 0.001 vs. Ctrl, # *p* < 0.05, ## *p* < 0.01, ### *p* < 0.001 vs. HG; ns, not significant.

Immunofluorescence analysis of α-SMA revealed that HG treatment markedly increased α-SMA signal intensity, indicating enhanced myofibroblast activation in CFs. In contrast, OMT treatment significantly reduced α-SMA fluorescence. Similarly, Met treatment effectively prevented the HG-induced increase in α-SMA intensity (Figure 6A,B), further confirming the suppression of myofibroblast activation. Wound healing assays demonstrated that HG exposure significantly enhanced CFs migratory capacity, whereas OMT treatment markedly suppressed cell migration. Consistently, the positive control Met also significantly inhibited CFs motility to a level similar to the high-dose OMT group (Figure 6C,D). Given the critical role of cell cycle regulation in cell proliferation and tissue remodeling [27], we next assessed the impact of OMT on CFs cell cycle progression. Quantitative analysis revealed that HG treatment significantly reduced the proportion of cells in the G0/G1 phase from 82.1% in the control group to 73.2% in the HG group (*p* < 0.01) while increasing the fraction of cells in the S phase from 5.2% to 11.1% (*p* < 0.001). Notably, treatment with OMT (0.8 mM) or the positive control Met effectively reversed this dysregulation, restoring G0/G1 phase occupancy to 82.5% and decreasing the S phase population to 5.9% compared to the HG group (both *p* < 0.001). These results indicate that OMT inhibits HG-induced aberrant proliferation by inducing G0/G1 arrest and suppressing S phase entry, mirroring the therapeutic effect observed with Met (Figure 6E).

### 2.4. The Construction of Transcription Factor Regulatory Networks and Molecular Docking

**Figure 7:**
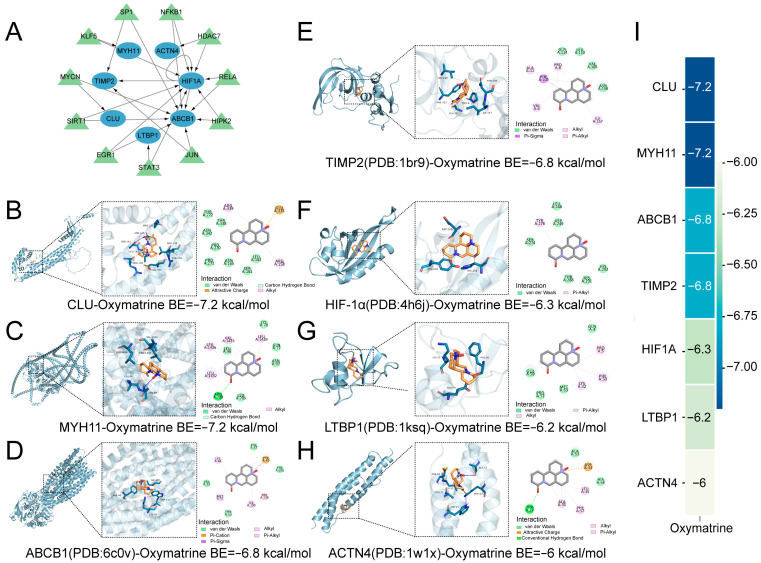
Regulatory Network of Transcription Factors and Core Genes and Molecular Docking Analysis (**A**) Regulatory network of core genes and transcription factors. Red nodes represent core genes, and yellow nodes represent transcription factors. The arrows between them indicate regulatory relationships. (**B**–**H**) Molecular docking results of OMT and key targets. (**B**) HIF-1α-OMT. (**C**) MYH11-OMT. (**D**) ABCB1-OMT. (**E**) TIMP2-OMT. (**F**) LTBP1-OMT. (**G**) ACTN4-OMT. (**H**) CLU-OMT. (**I**) Molecular docking heatmap. The heatmap visualizes the binding affinities between core target proteins and small molecule compounds. Color intensity represents the docking score or binding energy, with cooler colors indicating stronger binding affinity.

The 25 hub genes were submitted to the TRRUST database to identify associated transcription factors and construct transcriptional regulatory networks. This analysis yielded a regulatory network comprising 7 key genes (*HIF-1α*, *ACTN4*, *ABCB1*, *LTBP1*, *CLU*, *TIMP2*, and *MYH11*) along with their corresponding transcription factors (Figure 7A). To evaluate the potential interactions between OMT and these core targets, molecular docking analyses were performed, and the predicted binding modes were visualized (Figure 7B–H). The binding affinities between OMT and the hub targets are summarized in a heatmap (Figure 7I), in which color intensity reflects binding energy (kcal/mol), with lower values indicating stronger predicted interactions.

### 2.5. MD Simulation

**Figure 8:**
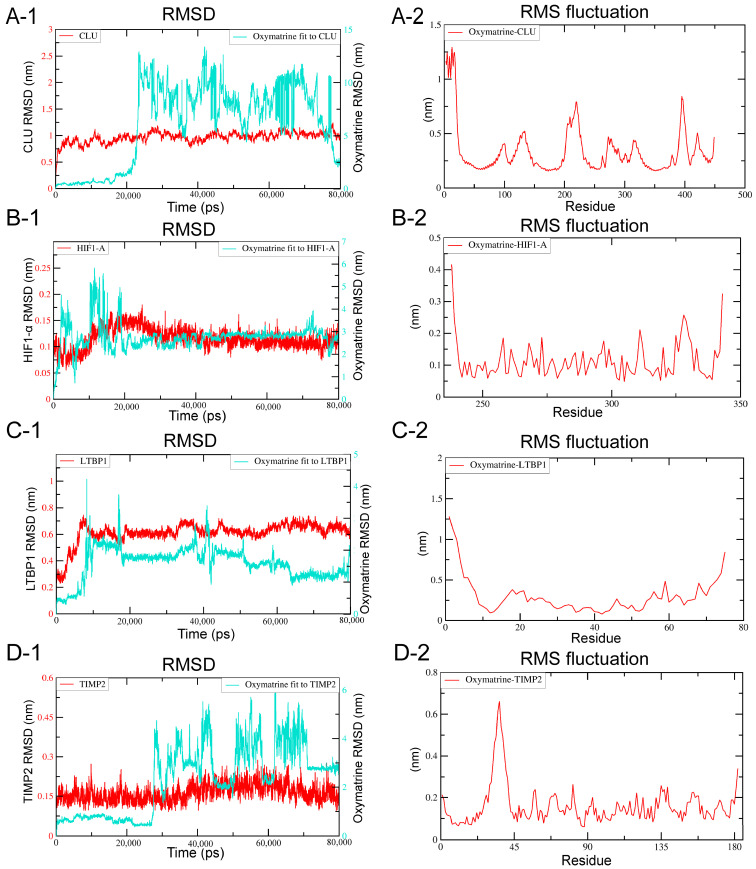
Molecular dynamics simulation analysis of OMT in complex with different protein targets (CLU, HIF-1α, LTBP1, and TIMP2). (**A1**–**D1**) RMSD plots of the protein backbones (red) and bound OMT ligand (cyan) over the 80 ns simulation. Following initial equilibration, the complexes generally maintained stable trajectories. (**A2**–**D2**) RMSF plots showing residue-level fluctuations of each protein target during the simulation. Relatively higher flexibility was mainly confined to the loop and terminal regions.

Binding energies derived from molecular docking represent static estimates and do not account for protein and ligand flexibility or solvent effects. To more comprehensively assess binding stability and functional relevance, representative complexes exhibiting reasonable binding poses and moderate docking scores—including HIF-1α, CLU, TIMP2 and LTBP1—were selected for MD simulations. MD simulations were performed to evaluate the structural dynamics of OMT in complex with HIF-1α, CLU, TIMP2, and LTBP1. As shown in the RMSD profiles (Figure 8(A1–D1)), the backbone Cα atoms of all four target proteins (red) and the OMT ligand (cyan) reached equilibrium after approximately 20 ns of simulation. The OMT-LTBP1 complex exhibited an average RMSD value of 0.60 ± 0.05 nm. RMSF analysis (Figure 8(A2–D2)) further showed that residue fluctuations were mainly localized to loop and terminal regions, whereas the core binding regions remained comparatively rigid throughout the simulation. In addition, radius of gyration (Rg) analysis (Figure S3(A1–D1)) confirmed that the global compactness of the protein structures remained constant, with Rg values for the LTBP1 complex fluctuating within a narrow range of 1.20–1.35 nm. Solvent-accessible surface area (SASA) analysis (Figure S3(A2–D2)) showed only modest fluctuations during the simulation, suggesting that OMT binding did not induce marked changes in solvent exposure and that the complexes maintained a relatively stable conformational state.

**Figure 9:**
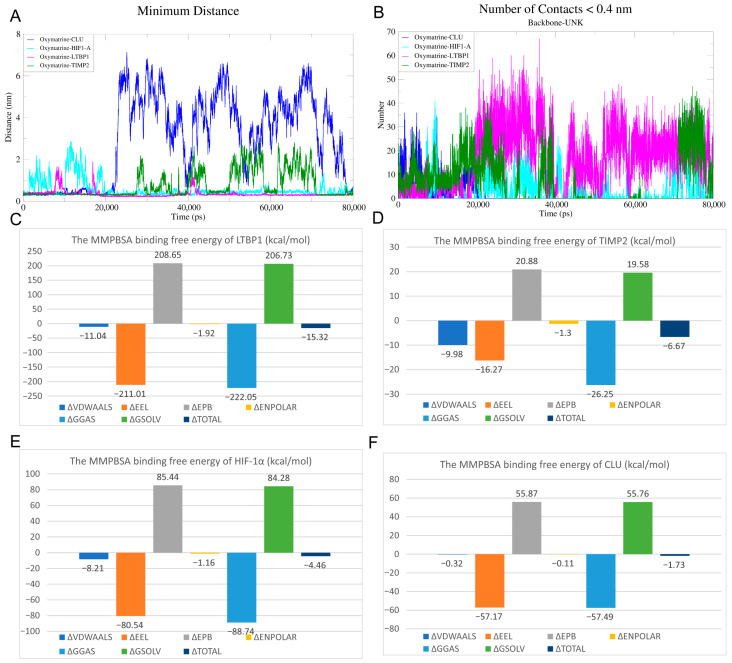
Interaction analysis and binding free energy evaluation of OMT with CLU, HIF-1α, LTBP1, and TIMP2. (**A**) Minimum distance plot showing the closest atomic distances between OMT and each target protein (CLU, HIF-1α, LTBP1, TIMP2) over an 80 ns simulation. A consistently low minimum distance indicates stable binding. (**B**) Number of contacts (<0.4 nm) between OMT and target proteins during the simulation. Higher contact numbers reflect stronger or more persistent interactions. (**C**–**F**) Binding free energy components of OMT with (**C**) LTBP1, (**D**) TIMP2, (**E**) HIF-1α, and (**F**) CLU, calculated by the MM/PBSA method. Energy contributions include van der Waals (VDW), electrostatic (ELE), polar solvation (PBSOL), and non-polar solvation (NPOLAR) energies.

To elucidate the binding preferences, the interaction behavior of OMT with the four target proteins was analyzed. Minimum distance analysis (Figure 9A) showed that the distance between OMT and the binding pocket of LTBP1 remained consistently stable, fluctuating around 0.2 nm throughout the simulation. Atomic contact analysis (defined as distance < 0.4 nm, Figure 9B) indicated that the OMT-LTBP1 complex exhibited the highest contact frequency compared to other groups. To quantitatively assess binding affinity, MM/PBSA calculations were performed (Figure 9C–F). OMT exhibited the lowest binding free energy (strongest affinity) for LTBP1 (−15.32 kcal/mol), followed by TIMP2 (−6.67 kcal/mol), HIF-1α (−4.46 kcal/mol), and CLU (−1.73 kcal/mol). Energy decomposition analysis revealed that van der Waals interactions and nonpolar solvation energy were the dominant favorable contributors to binding stability. Specifically, for the OMT–LTBP1 interaction, the van der Waals energy contribution was −11.04 kcal/mol, which, together with nonpolar solvation, effectively stabilized the complex.

### 2.6. OMT Attenuates Pathological Damage by Inhibiting the LTBP1/TGF-β/SMAD Signaling Pathway Under HG Conditions

**Figure 10:**
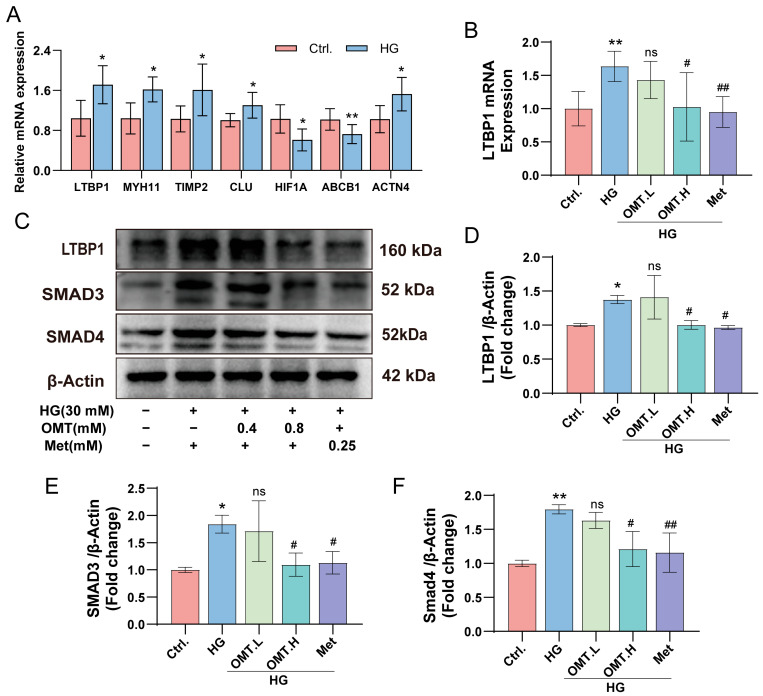
Effect of OMT on the expression of LTBP1/SMADs signaling pathway molecules in HG-induced cells. (**A**) The RT-qPCR validation of core gene expression in cardiac fibroblasts under HG conditions (*n* = 6). (**B**) Expression of LTBP1 under HG conditions following OMT treatment, detected using RT-qPCR (*n* = 6). (**C**–**F**) Protein expression level of LTBP1, SMAD3 and SMAD4 after OMT treatment. Data are presented as mean ± SD. * *p* < 0.05, ** *p* < 0.01 vs. Ctrl, # *p* < 0.05, ## *p* < 0.01 vs. HG; ns, not significant.

To experimentally validate the bioinformatic predictions, reverse transcription–quantitative PCR (RT-qPCR) was performed to assess the expression of selected hub genes in CFs exposed to HG conditions. Consistent with the in silico analysis, HG stimulation resulted in significant upregulation of LTBP1, MYH11, TIMP2, CLU and ACTN4, accompanied by downregulation of HIF-1α and ABCB1, relative to control cells (Figure 10A). Given the established role of LTBP1 as a key mediator of fibrotic processes, we next examined whether OMT could modulate its expression. Treatment of HG-stimulated CFs with OMT markedly attenuated the HG-induced increase in LTBP1 mRNA levels (Figure 10B). Similarly, the positive control effectively reversed the HG-induced upregulation of LTBP1, validating the reliability of our experimental model. Considering LTBP1 is a critical regulator of the TGF-β signaling cascade, we further assessed the expression of key downstream effectors of this pathway, SMAD3 and SMAD4. HG exposure significantly increased the protein levels of SMAD3 and SMAD4, indicating aberrant activation of the TGF-β pathway under hyperglycemic conditions. Notably, OMT treatment substantially suppressed the HG-induced upregulation of both SMAD proteins. Consistently, met treatment also inhibited the activation of these key TGF-β signaling components, exhibiting efficacy comparable to that of the high-dose OMT group (Figure 10C–F). Collectively, these results demonstrate that OMT mitigates HG-induced pathological responses, a process characterized by the downregulation of LTBP1 expression and the concurrent suppression of the TGF-β/SMAD signaling pathway, mirroring the protective effects observed with Met. These findings suggest a potential functional association between OMT-mediated LTBP1 modulation and the alleviation of myocardial fibrosis in DCM.

## 3. Discussion

DM represents a major global health challenge, with systemic complications that markedly increase the risk of cardiovascular disease. Among these, DCM is a distinct and severe complication that contributes substantially to the development of heart failure (HF) in patients with diabetes [28]. It emerges from the convergence of metabolic stress, neurohormonal activation, and maladaptive ECM remodeling. Although hyperglycemia is a defining trigger, the downstream molecular circuitry driving myocardial fibrosis remains incompletely resolved. In this study, we adopted a systems-to-mechanism strategy to interrogate the antifibrotic potential of OMT, integrating transcriptomic meta-analysis, structural modeling, and functional validation in HG-stimulated cardiac fibroblasts.

By integrating six independent Gene Expression Omnibus (GEO) datasets and rigorously eliminating batch-associated variance (Figure 1 and Figure 2), we defined a robust transcriptional signature shared between DM and CM. Intersection analysis yielded 91 Co-DEGs and 25 hub genes (Figure 4). Functional enrichment analyses based on GO and KEGG pathways revealed that these hub genes were significantly enriched in pathways implicated in DCM, including insulin resistance and insulin signaling [29]. These pathways are critically involved in the metabolic disturbances, impaired glucose utilization, sympathetic overactivation, myocardial remodeling and contractile dysfunction that characterize DCM [30]. These pathways are not isolated drivers but form a metabolically stressed signaling landscape that predisposes the myocardium to fibroblast activation and ECM accumulation. To translate these bioinformatic predictions into actionable experimental targets for pharmacological intervention with OMT, we prioritized candidates at the intersection of network centrality and ECM regulatory function, rather than selecting targets solely based on fold change. Consequently, LTBP1, TIMP2, and CLU—which occupy nodal positions within fibrotic and stress-response networks—were identified as mechanistically plausible mediators linking metabolic dysregulation to structural remodeling. This network-based prioritization provided the conceptual basis for our subsequent validation, specifically aiming to determine whether OMT could interrupt these maladaptive cascades by targeting these identified ECM-regulatory hubs.

HG exposure induced classical features of fibroblast activation, including enhanced proliferation, migration, α-SMA expression, and increased collagen synthesis (Figure 5 and Figure 6), mimicking specific aspects of diabetic fibrotic remodeling. OMT significantly attenuated these phenotypic transitions, restoring G0/G1 cell cycle distribution and reducing profibrotic protein expression. Importantly, these effects suggest that OMT does not merely suppress ECM gene transcription but interferes with fibroblast state transition. Fibroblast activation in DCM involves metabolic reprogramming, cytoskeletal remodeling, and autocrine amplification of profibrotic signals. The ability of OMT to modulate proliferation and migration implies upstream regulatory effects on signaling nodes governing cell fate decisions, rather than isolated downstream inhibition.

Structural modeling identified LTBP1 as the most energetically favorable predicted binding partner of OMT (Figure 9 and Figure 10), a finding functionally supported by reduced LTBP1 expression and downstream SMAD3/4 signaling in HG-treated cells (Figure 10). LTBP1 serves as a critical scaffold regulating TGF-β sequestration and activation within the ECM [31,32]. In the diabetic myocardium, excessive TGF-β activation amplifies SMAD-dependent transcription of collagen and α-SMA, reinforcing fibrotic progression.

Our findings support a model in which OMT dampens fibrotic amplification by limiting LTBP1-mediated facilitation of TGF-β signaling. However, this axis likely represents a major node rather than the sole mechanism. The enrichment of insulin and adrenergic signaling pathways in the hub gene network suggests that OMT may also mitigate metabolic stress-driven profibrotic signaling. Given the pleiotropic pharmacology of OMT—including reported anti-inflammatory and anti-oxidative properties—it is plausible that modulation of redox balance or inflammatory mediators indirectly constrains fibroblast activation. Moreover, although LTBP1 was selected as the primary target for in-depth validation in the present study, other candidate targets identified through the bioinformatic analysis may also contribute to the anti-fibrotic effects of OMT and warrant further investigation. Thus, LTBP1/TGF-β signaling may function as a convergence point integrating multiple upstream regulatory inputs influenced by OMT, supporting its role as a putative regulatory hub involved in fibrosis attenuation in the diabetic heart.

Nevertheless, several limitations must be acknowledged. First, the experimental validation was mainly restricted to HG-stimulated cardiac fibroblasts, which cannot fully recapitulate the multicellular interactions and complex pathological microenvironment of DCM in vivo. Therefore, further studies using diabetic animal models are required to evaluate the protective effects of OMT on cardiac fibrosis, remodeling, stiffness, and functional impairment at the whole-organism level. Our validation experiments were restricted to HG-stimulated cardiac fibroblasts and therefore do not capture the multicellular complexity of the diabetic myocardium. Computational docking, although informative, does not establish direct biochemical interaction, and future biophysical assays (ITC or SPR) are required to confirm binding kinetics and affinity. Furthermore, LTBP1 loss-of-function studies were not performed, limiting causal inference regarding its necessity in mediating OMT’s antifibrotic effects. In addition, although bioinformatic analysis identified multiple potentially relevant targets, the present study focused primarily on LTBP1, and the functional roles of other candidate targets remain to be experimentally clarified. Future studies should evaluate OMT in diabetic animal models to determine its impact on cardiac stiffness, remodeling, and functional parameters. Dissecting upstream regulators—including oxidative stress pathways, inflammatory mediators, and metabolic sensors—will clarify whether LTBP1 modulation represents a primary mechanism or a downstream consequence of broader cellular reprogramming. Collectively, our findings support a conceptual framework in which DCM fibrosis reflects coordinated network dysregulation linking metabolism, stress signaling, and ECM remodeling. Targeting ECM-associated regulatory hubs such as LTBP1 may therefore represent a promising strategy for interrupting maladaptive remodeling in diabetic heart disease.

## 4. Materials and Methods

### 4.1. Data Download and Processing

Gene expression profiles related to diabetes mellitus (DM) and cardiomyopathy (CM) were retrieved from the GEO database (https://www.ncbi.nlm.nih.gov/geo (accessed on 17 July 2024)). GEO datasets were independently searched using the keywords “diabetic” and “cardiomyopathy”. The inclusion criteria were as follows: (1) the organism was restricted to *Homo sapiens*; (2) the study design contained clearly defined disease and control groups; and (3) complete platform annotation files were available to ensure reproducible downstream analysis. Based on these criteria, six GEO datasets were included, comprising three DM-associated datasets (GSE15932, GSE95849, and GSE249102) and three CM-associated datasets (GSE21610, GSE42955, and GSE79962). The number of disease and control samples included from each dataset, together with the corresponding group definitions, is summarized in Table 1.

All data preprocessing and statistical analyses were performed in R (version 4.3.1). For each dataset, probes were annotated according to the corresponding platform annotation files, and expression values from multiple probes mapping to the same gene were averaged using the avereps function in the limma package. The distribution of expression values was examined, and datasets that had not been log-transformed were subjected to log2(x + 1) transformation. Subsequently, between-array normalization was performed using the normalizeBetweenArrays() function in limma. For integrative analysis, only genes shared across datasets within each disease category were retained to construct merged expression matrices, and each dataset was treated as an independent technical batch. Batch effects were corrected using the ComBat() function in the sva package with the par.prior = TRUE, implementing a parametric empirical Bayes framework. The effectiveness of batch correction was visually assessed by principal component analysis (PCA) before and after correction.

### 4.2. Differential Gene Expression Analysis

Differentially expressed genes (DEGs) were identified separately for the DM-related and CM-related merged, batch-corrected expression matrices by comparing disease and control groups using the limma package. After fitting linear models with lmFit and applying empirical Bayes moderation with eBayes, genes were ranked using the topTable function with Benjamini–Hochberg false discovery rate (FDR) adjustment. DEGs were screened using the thresholds of adjusted *p* < 0.10 and |log2 fold change| > 0.264 (equivalent to fold change > 1.2). This relatively permissive threshold was adopted to capture moderate but potentially biologically meaningful transcriptional alterations in complex chronic diseases. The results were visualized using volcano plots generated with ggplot2 and heatmaps generated with pheatmap. The intersection of DEGs identified from the DM-related and CM-related analyses was defined as Co-DEGs, which were considered potential candidate genes associated with DCM. Functional enrichment analysis of Co-DEGs was performed using the clusterProfiler package. GO enrichment analysis was conducted using enrichGO with OrgDb = org.Hs.eg.db and ont = “ALL”, andKEGG pathway analysis was conducted using enrichKEGG with organism = “hsa”. For enrichment analyses, *p* < 0.05 was considered statistically significant unless otherwise specified.

### 4.3. Construction of the PPI Network and Identification of Hub Genes

The Co-DEGs identified through differential expression analysis were entered into the STRING (version 11.5; http://string-db.org; accessed on 8 July 2024) to build the PPI network. To retain a comprehensive interaction landscape, a low confidence interaction score (minimum required interaction score = 0.15) was applied, allowing the inclusion of potentially relevant but weakly supported interactions. The resulting PPI network was exported in TSV format and visualized using Cytoscape 3.10.0. Hub genes were identified using the CytoHubba plugin. Among several available topological algorithms, degree centrality was selected because it reflects the number of direct interactions of each node and is commonly used to identify key regulatory genes in disease-associated networks. The top 25 genes ranked by degree value were defined as hub genes, which provided a balance between network representativeness and biological interpretability.

### 4.4. GeneMANIA Analysis, GO Analysis, and KEGG Pathway Analysis of the Key Hub Genes

The identified hub genes were further analyzed using the GeneMANIA database (https://genemania.org/; accessed on 8 July 2024) to predict functionally associated genes and construct an extended gene interaction network [33]. The network was generated for *Homo sapiens* based on diverse interaction categories, including co-expression, physical interactions, genetic interactions, shared protein domains, co-localization, and predicted interactions. The weighting method was set to ‘automatically selected weighting method’ to maximize connectivity based on the query list. Subsequently, GO and KEGG pathway enrichment analyses were performed as described in Section 4.2.

### 4.5. Transcription Factor Regulation of Core Genes

Regulatory factor information for specific genes was retrieved from the TRRUST database. All analyses were based on previously downloaded TRRUST data. Cytoscape was then used to construct and visualize the gene–transcription factor regulatory network.

### 4.6. Molecular Docking

The three-dimensional structures of the target receptors were primarily retrieved from the RCSB Protein Data Bank (RCSB PDB)database (https://www.rcsb.org/; accessed on 2 March 2025), prioritizing high-resolution human protein models. The selected crystal structures included: ABCB1 (PDB ID: 6C0V), TIMP2 (PDB ID: 1BR9), HIF-1α (PDB ID: 4H6J), LTBP1 (PDB ID: 1KSQ), and ACTN4 (PDB ID: 1W1X). For targets lacking experimental crystal structures (CLU and MYH11) or with incomplete regions, AlphaFold 2.3.1 was used to predict and supplement the missing parts. Predicted regions with a confidence score predicted Local Distance Difference Test (pLDDT) above 90 were selected to replace incomplete segments, ensuring overall structural integrity. The chemical structure of OMT was obtained from the PubChem database, and the SDF file was converted to PDBQT format using Open Babel 2.3.2 [34]. Prior to docking, all receptor structures were preprocessed using AutoDock Tools 1.5.7 and PyMOL 3.0. This process involved the removal of water molecules, addition of hydrogen atoms, merging of non-polar hydrogens, and assignment of Gasteiger charges. The grid box dimensions were set to 126 × 126 × 126 Å^3^ with a spacing of 1 Å, indicating blind docking. Molecular docking simulations were conducted using AutoDock Vina 1.5.7 [35] with the exhaustiveness parameter set to 20. A binding affinity lower than −5 kcal/mol was considered to indicate significant binding activity. Finally, binding modes and interactions were visualized using PyMOL 3.0.

### 4.7. MD Simulation

MD simulations were performed using GROMACS 2022.3 (GROMACS development teams, Royal Institute of Technology and Uppsala University, Sweden). The CHARMM36 force field (charmm36-jul2022.ff) was selected for protein modeling due to its proven reliability in accurately describing protein conformational dynamics and protein–ligand interactions in aqueous environments. Ligand force field parameters were generated using the CHARMM General Force Field (CGenFF) (https://app.cgenff.com/) web application (SilcsBio, LLC, Baltimore, MD, USA; accessed on 4 June 2026), which provides automated atom typing, charge assignment, and bonded parameters compatible with the CHARMM36 force field. The resulting parameter stream files were converted into GROMACS-compatible topology files and incorporated into the system topology. Prior to simulation, the complexes were solvated in a cubic box using the TIP3P water model, and sodium and chloride ions were added to neutralize the system and maintain a physiological salt concentration (0.15 M). The systems were first subjected to energy minimization using the steepest descent algorithm to eliminate steric clashes and unfavorable contacts. Subsequently, systems were equilibrated under NVT and NPT ensembles for 100 ps each with a 2 fs time step, while applying positional restraints to the protein and ligand. Temperature was maintained at 300 K using the V-rescale thermostat, and pressure was controlled at 1 bar using the Parrinello–Rahman barostat (during the NPT phase). All bonds involving hydrogen atoms were constrained using the LINCS algorithm, enabling the use of a 2 fs integration step. Nonbonded interactions were treated using the Verlet cutoff scheme, with Particle Mesh Ewald (PME) for long-range electrostatic interactions and a force-switch modifier for van der Waals interactions. Periodic boundary conditions were applied in all three dimensions. Following equilibration, production MD simulations were carried out for 80 ns. The convergence of backbone root-mean-square deviation (RMSD) and potential energy confirmed that this duration was sufficient to achieve conformational stability. Consequently, only the equilibrated trajectory segments were utilized for subsequent analyses, with coordinates saved every 10 ps. Global structural stability and residue-level flexibility were characterized using RMSD and Root-Mean-Square Fluctuation (RMSF), respectively. Binding free energies were calculated using the MM/PBSA method implemented in the gmx_MMPBSA module (version 1.6.3; open-source module for GROMACS based on AMBER’s MMPBSA.py) [36]. The binding free energy was defined as the difference between the total free energy (molecular mechanics potential plus solvation energy) of the complex and the sum of those for the unbound receptor and ligand.

### 4.8. Cell Culture and Treatment

Adult Sprague–Dawley (SD) rats were used for breeding to obtain neonatal rats. All animal procedures were conducted in accordance with institutional guidelines and were approved by the Animal Care and Welfare Committee of Guizhou Medical University (Approval No. 2305270; approval date: 11 May 2023). Primary cardiac fibroblasts (CFs) were isolated from the hearts of 1–2-day-old neonatal SD rats by enzymatic digestion and cultured in complete Dulbecco’s Modified Eagle Medium (DMEM, Gibco, Grand Island, NY, USA) containing 5.5 mM D-glucose, 10% fetal bovine serum (FBS, TransSerum, Brisbane, Australia) and 1% penicillin–streptomycin, as previously described [37,38]. CFs were dissociated using 0.25% trypsin-EDTA (Gibco, USA) and expanded to passage 2. The purity of the isolated CFs was verified to be >98% by immunofluorescence staining for vimentin prior to use in subsequent experimental treatments. The concentrations of OMT (0.4 and 0.8 mM) were selected based on preliminary cell viability assays, which demonstrated that these doses were non-cytotoxic and optimal for intervention. Cells were then randomly assigned to five groups: control (5.5 mM glucose), HG (30 mM glucose), HG + OMT-L (0.4 mM), HG + OMT-H (0.8 mM) and HG + metformin (Met, 0.25 mM). Prior to stimulation, CFs were serum-starved for 24 h to achieve synchronisation. Cells in the treatment groups were pretreated with OMT or Met for 2 h, followed by exposure to HG medium containing the indicated concentrations of OMT or Met for 24 h.

### 4.9. CCK-8 Assay

The viability and proliferative capacity of CFs were assessed utilizing the Cell Counting Kit-8 (CCK-8, Solarbio, Beijing, China). Cells were seeded into 96-well plates at a density of 3 × 10^4^ cells per well and cultured for 24 h. To systematically determine the optimal experimental conditions and evaluate the effects of OMT, three distinct assays were performed: first, to identify the optimal glucose concentration for inducing proliferation, CFs were exposed to a glucose gradient (5.5, 15, 20, 25, 30, 35, 40, 45, and 50 mM) for 24 h; second, to evaluate the cytotoxicity and safety profile of OMT, cells were treated with increasing concentrations of OMT (0.001–1.6 mM) in normal culture medium; and third, to assess the therapeutic effect of OMT on HG-induced proliferation, cells were co-treated with the selected HG concentration (30 mM) and various concentrations of OMT (0.2–1.4 mM). For all assays, six parallel replicates were established for each condition. Following the respective treatments, 10 μL of CCK-8 reagent was added to each well containing 100 μL of culture medium. The plates were then incubated at 37 °C for 3 h, after which optical density (OD) values were measured at 450 nm using a Varioskan™ LUX multimode microplate reader (Thermo Fisher Scientific, Waltham, MA, USA).

### 4.10. RT-qPCR

Total RNA was extracted from CFs using the RNeasy Isolation Reagent Kit (Vazyme, Nanjing, China) and reverse-transcribed into cDNA with the EasyScript^®^ gDNA Removal and cDNA Synthesis Kit (TransGen Biotech, Beijing, China). The resulting cDNA served as the template for quantitative RT-qPCR, which was performed using PerfectStart^®^ Green qPCR SuperMix (TransGen Biotech Co., Ltd., Beijing, China) on a Bio-Rad CFX Manager 3.1 System (Bio-Rad Laboratories, Hercules, CA, USA). Relative mRNA expression levels were calculated using the 2^−ΔΔCt^ method [39], with β-actin employed as the internal reference gene. Primer sequences (Invitrogen, Shanghai, China) are listed in Table 2.

### 4.11. Western Blot (WB) Analysis

Total protein was extracted from CFs using RIPA lysis buffer (Solarbio, Beijing, China), and protein concentrations were determined with a bicinchoninic acid (BCA) assay kit (Solarbio, Beijing, China). Protein samples were denatured by heating at 100 °C for 10 min in loading buffer, separated by 10% SDS–PAGE, and subsequently transferred onto 0.45 μm polyvinylidene fluoride (PVDF) membranes. Membranes were blocked with 5% bovine serum albumin (BSA) for 2 h at room temperature and incubated overnight at 4 °C with the following primary antibodies: anti-collagen I (1:1000, 14695-1-AP), anti-collagen III (1:1000, 22734-1-AP), anti-fibronectin (1:1000, 15613-1-AP), anti-α-smooth muscle actin (α-SMA, 1:1000, 14395-1-AP), anti-Smad3 (1:1000, 66515-1-Ig), anti-Smad4 (1:1000, 10231-1-AP) and anti-latent transforming growth factor-β-binding protein 1 (LTBP1, 1:500, 85820-1-RR) (all from Proteintech, Wuhan, China). Following incubation with the appropriate horseradish peroxidase-conjugated secondary antibodies for 2 h at room temperature, protein signals were visualized using enhanced chemiluminescence (ECL, Xinsaimei Biotechnology, Suzhou, China). Immunoreactive bands were detected with a ChemiDoc XRS+ imaging system (Bio-Rad Laboratories, Hercules, CA, USA), and band intensities were quantified using Image Lab 4.0 software (Bio-Rad Laboratories, Hercules, CA, USA).

### 4.12. Wound Healing Assay

CFs were seeded into six-well plates and cultured to near confluence. A uniform linear scratch was generated in the center of each well using a sterile 200 μL pipette tip (Thermo Fisher Scientific, Waltham, MA, USA). After gentle washing to remove detached cells, CFs were serum-starved and pretreated with OMT or Met for 2 h, followed by exposure to HG medium containing the indicated concentrations of OMT or Met (supplemented with 0.5% FBS). Images of the wounded areas were acquired at 0 and 24 h using an inverted microscope, and wound closure was quantified with ImageJ software to evaluate CFs migratory capacity.

### 4.13. Immunofluorescence Analysis

CFs were fixed with 4% paraformaldehyde at room temperature for 20 min and permeabilized with 0.5% Triton X-100 for 15 min. Cells were then incubated overnight at 4 °C with primary antibodies against vimentin (1:500, 10366-1-AP, Proteintech, Wuhan, China) and α-SMA (1:500; 14395-1-AP, Proteintech, Wuhan, China). After washing, CFs were incubated with Alexa Fluor-conjugated secondary antibodies (1:1000) for 2 h at room temperature in the dark, followed by nuclear counterstaining with DAPI for 5 min. Fluorescence images were acquired using a Nikon Eclipse C1 microscope (Nikon, Tokyo, Japan), and fluorescence intensity was quantified as mean fluorescence intensity using ImageJ v1.52a software.

### 4.14. Cell Cycle Analysis

After 24 h of treatment according to the experimental design, cells were harvested and fixed in 70% cold ethanol at 4 °C. Following washing with PBS to remove residual fixative, cells were incubated with the working solution from the cell cycle detection kit (RNase A:propidium iodide (PI) = 1:9, KGA9101-20, KeyGEN BioTECH, Nanjing, China) for 30–60 min in the dark. Cell cycle distribution was subsequently analyzed by detecting red fluorescence using a NovoCyte flow cytometer (Agilent Technologies, Santa Clara, CA, USA) with excitation at 488 nm. Data quantification and analysis were performed using NovoExpress software, version 1.0.13 (Agilent Technologies, Santa Clara, CA, USA).

### 4.15. Statistical Analysis

For bioinformatic analyses, data preprocessing and statistical calculations were conducted in R (version 4.3.1), mainly using the limma (version 3.58.1), sva (version 3.50.0), clusterProfiler (version 4.10.1), ggplot2 (version 4.0.1), and pheatmap (version 1.0.13) packages. Probe summarization, normalization, batch-effect correction, and differential expression analysis were performed as described above. For DEG identification, *p* values were adjusted for multiple testing using the Benjamini–Hochberg false discovery rate method, and genes with adjusted *p* < 0.10 and |log2 fold change| > 0.264 were considered differentially expressed. The effectiveness of batch-effect removal was visually assessed by PCA before and after correction. Separate formal tests of normality of residuals and homogeneity of variance were not performed for the bioinformatic analyses; instead, standard preprocessing, normalization, batch correction, and PCA-based visual inspection were used to improve data comparability.

For the experimental validation data, results are presented as mean ± SD from at least three independent experiments, unless otherwise stated. Statistical analyses were performed using GraphPad Prism 8.0. Comparisons between two groups were conducted using an unpaired Student’s *t*-test, whereas comparisons among multiple groups were performed using one-way ANOVA followed by Tukey’s post hoc test. As formal tests for normality and homogeneity of variance were not performed, this was considered a methodological limitation, and the results were interpreted with caution. A two-sided *p* < 0.05 was considered statistically significant for the experimental analyses.

## 5. Conclusions

In summary, our findings integrate transcriptomic network analysis with in vitro functional validation to suggest that OMT attenuates HG-induced fibroblast activation, at least in part, through modulation of the LTBP1/TGF-β/SMAD axis. These results identify LTBP1 as a putative regulatory node within the fibrotic network of DCM. In addition, other candidate targets revealed by the bioinformatic analysis may also be involved in the anti-fibrotic effects of OMT and deserve further investigation. Overall, our findings provide mechanistic insight into the anti-fibrotic action of OMT and suggest its potential therapeutic value, which should be further validated in diabetic animal models to assess its efficacy and safety in a systemic context.

## Figures and Tables

**Table 1:** Summary of Gene Expression Omnibus (GEO) datasets included in this study.

No	GEO Accession	Platform	Organism	Group Comparison	Sample Size (Control/Case)	PMID
1	GSE15932	GPL570	*Homo sapiens*	healthy control vs. diabetes mellitus	8/8	-
2	GSE95849	GPL22448	*Homo sapiens*	CN vs. DM	6/6	28900628
3	GSE249102	GPL10332	*Homo sapiens*	CTRL vs. DM2	4/4	38801463
4	GSE21610	GPL570	*Homo sapiens*	NF vs. VAD	8/30	20460602
5	GSE42955	GPL6244	*Homo sapiens*	control hearts vs. cardiomyopathy	5/24	24339868
6	GSE79962	GPL6244	*Homo sapiens*	non-failing donors vs. IHD/DCM	11 vs. 11/9	28067713

**Table 2:** Primer sequences used for Reverse Transcription-Quantitative PCR (RT-qPCR).

Gene	Primer	Sequence (5′–3′)
*HIF-1α*	forward	CAGACAAAGCTCATCCAAGGAG
*HIF-1α*	reverse	TGCAGTAACGTTCCAATTCCT
*MYH11*	forward	CAGTAGCAGATCTGGGACCAC
*MYH11*	reverse	CGAAGCCCTGCTTCTCTGAA
*ABCB1*	forward	TGAACTGTGACCATGCGAGAT
*ABCB1*	reverse	GTCTCTGAAGACTCTAAAATGGACT
*TIMP2*	forward	TGAGGTCTCATGCTGAGGGCAG
*TIMP2*	reverse	GGCATAACGCGACAGAGAGATG
*LTBP1*	forward	CCATAGCACCTCAGCAGCAC
*LTBP1*	reverse	GTGGAGGCCAACTGGGTG
*ACTN4*	forward	GGCACAGACCTGAGCTGATT
*ACTN4*	reverse	GTGTTCACGATGTCCTCAGC
*CLU*	forward	GGCTTTCCCGGAAGTGTGTA
*CLU*	reverse	AAACTCCTCTAGCTGGCGAC
*β-Actin*	forward	TGTCACCAACTGGGACGATA
*β-Actin*	reverse	GGGGTGTTGAAGGTCTCAAA

## Data Availability

The datasets analyzed for this study can be found in the Gene Expression Omnibus (GEO, https://www.ncbi.nlm.nih.gov/geo/, accessed on 17 July 2024). GSE15932: Yulian Wu|2012|Blood biomarkers of pancreatic cancer associated diabetes identified by peripheral blood-based gene expression profiles| https://www.ncbi.nlm.nih.gov/geo/query/acc.cgi?acc=GSE15932| NCBI Gene Expression Omnibus, GSE15932; GSE95849: Lin Luo, Wen-Hua Zhou, Jiang-Jia Cai, Mei Feng, Mi Zhou, Su-Pei Hu, Jin Xu, Lin-Dan Ji|2017|Transcriptional profiling of diabetic peripheral neuropathy patients, diabetic patients, and healthy participants| https://www.ncbi.nlm.nih.gov/geo/query/acc.cgi?acc=GSE95849| NCBI Gene Expression Omnibus, GSE95849; GSE249102: Elena Jaime-Sánchez, Edgar E Lara-Ramírez, Juan Ernesto López-Ramos, Elsy Janeth Ramos-González, Ana Laura Cisneros-Méndez, Juan José Oropeza-Valdez, Roberto Zenteno-Cuevas, Gerardo Martínez-Aguilar, Yadira Bastian, Julio Enrique Castañeda-Delgado, Carmen Judith Serrano, José Antonio Enciso-Moreno |2024| Whole RNA profiling of Tuberculosis-diabetes comorbidity linked to diabetes with poor glycemic control | https://www.ncbi.nlm.nih.gov/geo/query/acc.cgi?acc=GSE249102| NCBI Gene Expression Omnibus, GSE249102; GSE21610 Patrick Schwientek, Peter Ellinghaus, Sonja Steppan, Donatella D’Urso, Michael Seewald, Astrid Kassner, Ramona Cebulla, Sebastian Schulte-Eistrup, Michiel Morshuis, Daniela Röfe, Aly El Banayosy, Reiner Körfer, Hendrik Milting|2010| Gene expression analysis in non-failing and failing myocardium pre and post pulsatile and non-pulsatile VAD support| https://www.ncbi.nlm.nih.gov/geo/query/acc.cgi?acc=GSE21610|NCBI Gene Expression Omnibus, GSE21610; GSE42955: Maria Micaela Molina-Navarro, Esther Roselló-Lletí, Ana Ortega, Estefanía Tarazón, Manuel Otero, Luis Martínez-Dolz, Francisca Lago, José Ramón González-Juanatey, Francisco España, Pablo García-Pavía, José Anastasio Montero, Manuel Portolés, Miguel Rivera|2013|Expression data from human heart| https://www.ncbi.nlm.nih.gov/geo/query/acc.cgi?acc=GSE42955| NCBI Gene Expression Omnibus, GSE42955; GSE79962: Scot J Matkovich, Belal Al Khiami, Igor R Efimov, Sarah Evans, Justin Vader, Ashwin Jain, Bernard H Brownstein, Richard S Hotchkiss, Douglas L Mann|2016|Widespread downregulation of cardiac mitochondrial and sarcomeric genes in patients with sepsis| https://www.ncbi.nlm.nih.gov/geo/query/acc.cgi?acc=GSE79962| NCBI Gene Expression Omnibus, GSE79962.

## Supplementary Materials

The following supporting information can be downloaded at: https://www.mdpi.com/article/10.3390/ijms27083481/s1.

## Abbreviations

The following abbreviations are used in this manuscript:

OMT	Oxymatrine
DCM	Diabetic cardiomyopathy
CM	Diabetes mellitus
ECM	Extracellular matrix
CFs	Cardiac fibroblasts
HIF-1α	Hypoxia-Inducible Factor 1 Alpha
ACTN4	Actinin Alpha 4
ABCB1	ATP Binding Cassette Subfamily B Member 1
CLU	Clusterin
TIMP2	Tissue Inhibitor of Metalloproteinases 2
MYH11	Myosin Heavy Chain 11
DEGs	Differentially Expressed Genes
HG	High Glucose
Met	Metformin
