# Supplementary material for: Screening and Validation of LTBP1 as a Key Target of Oxymatrine in Inhibiting Cardiac Fibroblast Differentiation Under High Glucose Conditions: In Vitro and Bioinformatic Studies
Source: Int J Mol Sci. 2026 Apr 13;27(8):3481. doi: 10.3390/ijms27083481 (PMC13117001; doi:10.3390/ijms27083481)
Supplement: Supplementary file 1 [file ijms-27-03481-s001.zip › ijms-4202188-supplementary.pdf]

Supplementary Figure S1

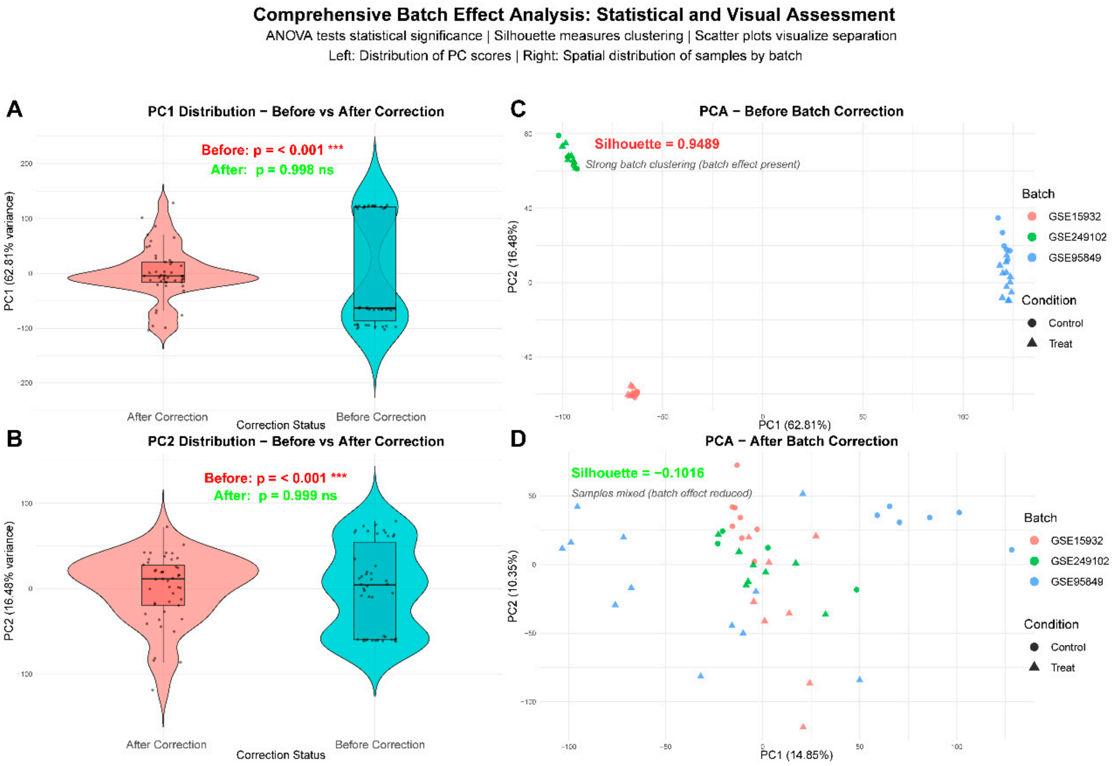

Supplementary Figure S1 Comprehensive assessment of batch effect correction in DM-related datasets. Batch effects among datasets GSE15932, GSE95849, and GSE249102 were corrected using the R package sva. **(A, B)** Violin plots with embedded boxplots showing the distribution of PC1 (A) and PC2 (B) scores before and after batch correction. Statistical significance was assessed by ANOVA; p values are indicated. **(C, D)** PCA plots displaying sample distribution colored by dataset origin (GSE15932, red; GSE249102, green; GSE95849, blue) and shaped by condition (Control, circle; Treat, triangle) before (C) and after (D) correction. Silhouette coefficients quantifying batch clustering are shown; values approaching 1 indicate strong batch separation, whereas values near 0 or negative values indicate effective batch mixing. ns, not significant.

Supplementary Figure S2

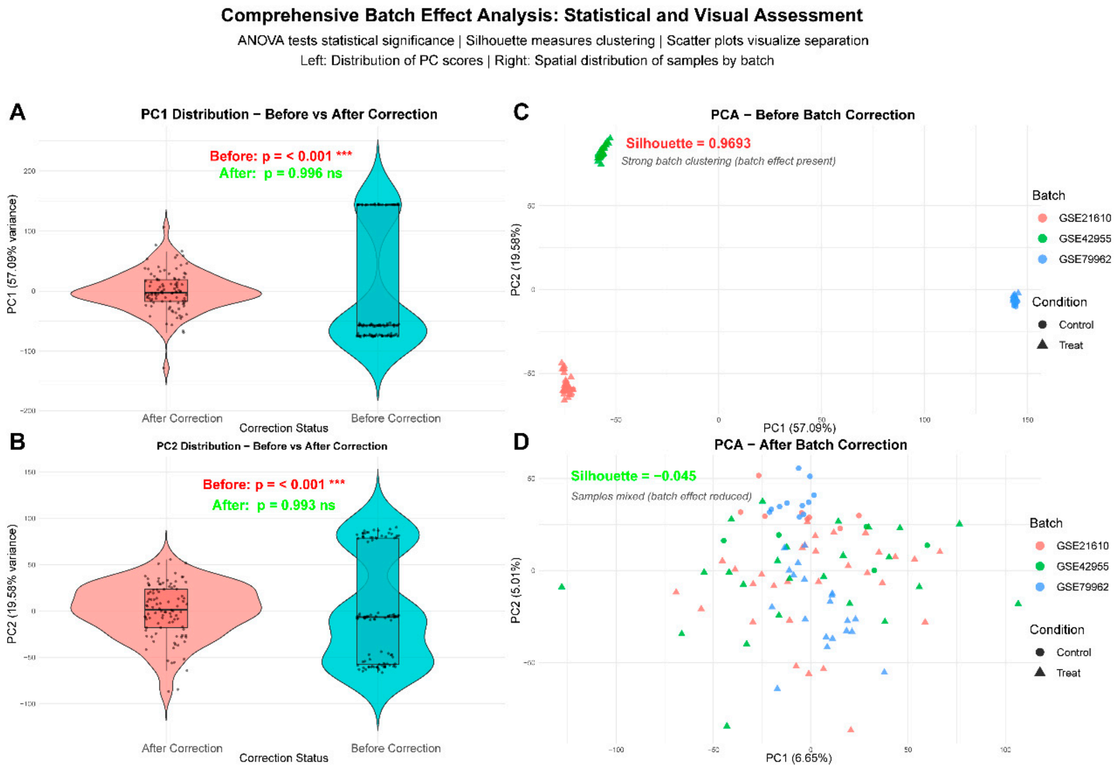

Supplementary Figure S2 Comprehensive assessment of batch effect correction in CM-related datasets. Batch effects among datasets GSE21610, GSE42955, and GSE79962 were corrected using the R package sva. **(A, B)** Violin plots with embedded boxplots showing the distribution of PC1 (A) and PC2 (B) scores before and after batch correction. Statistical significance was assessed by ANOVA; p values are indicated. **(C, D)** PCA plots displaying sample distribution colored by dataset origin (GSE21610, red; GSE42955, green; GSE79962, blue) and shaped by condition (Control, circle; Treat, triangle) before (C) and after (D) correction. Silhouette coefficients quantifying batch clustering are shown; values approaching 1 indicate strong batch separation, whereas values near 0 or negative values indicate effective batch mixing. ns, not significant.

Supplementary Figure S3

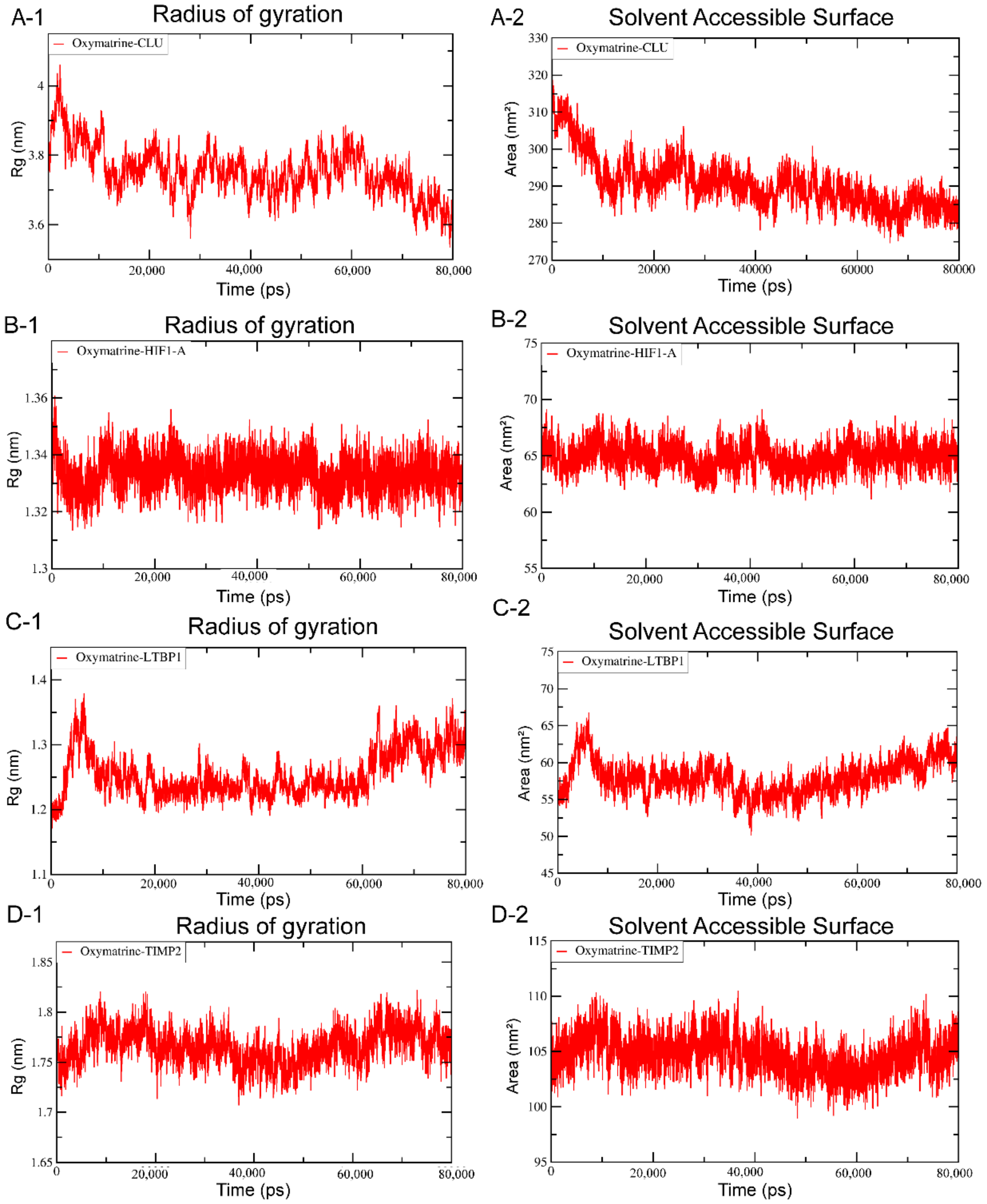

Supplementary Figure S3 Complementary molecular dynamics simulation parameters for OMT in complex with different protein targets (CLU, HIF-1α, LTBP1, and TIMP2) (A1–D1) SASA profiles showing the solvent exposure of each complex during the 80 ns simulation. **(A2–D2)** Radius of gyration (Rg) plots showing the overall compactness of each protein structure throughout the simulation.
